# Isolated Flexor Digitorum Profundus Injuries in Flexor Zone II of the Hand: A Report of Five Cases

**DOI:** 10.7759/cureus.34360

**Published:** 2023-01-29

**Authors:** Ahmed F Alkandari, Abrar Alawadhi, Fatma A Alawadhi, Waleed M Renno

**Affiliations:** 1 Department of Surgery, Al-Adan Hospital, Ahmadi, KWT; 2 Department of Anatomy, Kuwait University, Jabriya, KWT

**Keywords:** case report, tendon cut, isolated fdp injury, flexor digitorum profundus, flexor digitorum superficialis, camper's chiasma, no man's land, the critical zone, flexor zone ii, traumatic hand injury

## Abstract

Injuries of the hand's flexor tendons carry a poor prognosis, mainly if they are in zone II (also called 'the critical zone' or 'no man's land'). The superficial tendon in this zone ends by bifurcating and attaching to the sides of the middle phalanx, exposing the deep tendon that attaches to the distal phalanx. Thus, trauma to this zone may result in a complete cut to the deep tendon while the superficial one remains intact. The lacerated tendon, in turn, would be retracted proximally to the palm making it difficult to be found during wound exploration. The complex anatomy of the hand, particularly that of the flexor zones, may contribute to the misdiagnosis of a tendon injury. We report five cases of an isolated cut of the flexor digitorum profundus (FDP) tendon after traumatic injury to the flexor zone II of the hand. The mechanism of injury of each case is reported together with a clinical approach that guides ED physicians toward diagnosing flexor tendon injuries in hand. In cut wounds involving the flexor zone II of the hand, it should be not surprising to find that the deep tendon (FDP) is completely lacerated without an injury to the superficial one (FDS). Therefore, we conclude that a systematic examination approach for traumatic hand injuries is essential to ensure the proper assessment. Understanding the mechanism of injury, performing a systemic examination approach, and having basic anatomical knowledge of flexor tendons of the hand are essential to identifying tendon injuries, anticipating complications, and providing adequate healthcare.

## Introduction

Traumatic hand injuries

Traumatic hand injuries are common and account for 20% of all hospital visits to emergency departments (EDs) [1] while the highest incidence occurs in middle-aged individuals and males [2]. Most injuries are work-related and accidentally induced by sharp objects [3]. The location of the injury is related to hand dominance and the mechanism of injury [4]. In adults, hand injuries usually involve the non-dominant hand due to its supportive role while the dominant hand performs a knife-cutting task. However, injury to the dominant hand can also occur due to handling or catching a falling sharp object [3], whereas, in children, they usually involve the dominant hand due to directly manipulating a sharp (knife) or fragile (glass) object [5]. Injury to the hands' flexor tendons can economically affect the patient, mainly if it is confined to his dominant hand [3].

Flexor tendon injuries

Complications following flexor tendon repair are categorized into early and late stages. Early complications include hematoma, infection, dehiscence, tendon or pulley rupture, and the tendon's triggering in the sheath [6]. Late complications include tendon adhesions, rupture after mobilization, flexion contracture, lumbrical plus deformity, and bowstringing [7]. Compared to the extensors, flexor tendon injuries have a poorer prognosis [8]. This is because the palmar aspect of the flexor tendon has an inadequate blood supply compared to the dorsal one, which affects tendon healing [9]. The fibrous-osseous pulley system also contributes to the poorer prognosis of flexor tendon injuries since injuries to the fibrous-osseous sheath will result in profound adhesions affecting the tendon function [6]. Moreover, concomitant nerve and vessel injuries are common due to their anatomical proximity [3], which can affect wound healing and result in poor outcomes.

Based on therapeutic and rehabilitation perspectives, the flexor tendons of the hand are divided into five specific zones. Flexor zone II is called 'the critical zone' or 'No man's land' because of its complicated anatomy [10], and tendon injuries in this zone carry the worst prognosis [7]. The complex anatomy of the hand, particularly that of the flexor zones, may contribute to the misdiagnosis of a tendon injury. Also, the unplanned extension of the surgical wound can adversely affect its outcome [11]. Such injuries, therefore, require detailed examination and appropriate referral [12,13]. In cut wounds involving the flexor zone II of the hand, it should be not surprising to find that the deep tendon is completely lacerated without an injury to the superficial one. Although deep cut wounds in the flexor zone II usually involve both tendons [14], laceration of the deep tendon (FDP) without affecting the superficial one (FDS) can rarely occur. This is because the superficial tendon bifurcates and becomes attached to the middle phalanx, allowing the deep tendon to pass underneath it and become attached to the distal phalanx. We herein report five cases of an isolated injury to the FDP tendon confined to flexor zone II.

This article was previously presented as a meeting abstract at the 25th Health Sciences Center (HSC) Poster Conference on March 16-18, 2022.

## Case presentation

Case I

In an attempt to cut fruits by himself, a seven-year-old boy injured his left hand with a knife. Three lacerations were located on the palmar aspect of the middle phalanges of his three middle fingers. Upon examination, he could flex his fingers at the proximal and distal IP joints, except the ring finger at the distal IP joint. Surgical exploration of the ring finger revealed intact FDS tendon slips, with the FDP tendon being retracted due to laceration.

Case II

Like the earlier case, a 33-year-old male accidentally injured his left hand with a knife while slicing fruits. He had three cut wounds on the palmar aspect of the middle phalanges of his three middle fingers. Examination showed an ability to flex the fingers at their proximal and distal IP joints but not the ring finger at the distal IP joint. Exploration of the ring finger showed an intact FDS tendon and a retracted FDP tendon.

Case III

A 24-year-old male accidentally stabbed the palm of his left index while separating frozen burger slices with a knife’s tip. A laceration was confined to the palm of the left hand in zone II over the middle phalanx of the index finger. The patient could flex all fingers at the proximal and distal IP joints but could not flex the index finger at the distal IP joint. Surgical exploration of the index showed an intact FDS tendon but a retracted FDP tendon.

Case IV

A 44-year-old male received a work-related injury to his hand while operating with sharp metals. The damage was induced by a sharp aluminum edge, causing a skin laceration to the palm of his dominant right hand's little finger. While examination, the patient could flex his fingers at the proximal and distal IP joints except for the little finger at the distal IP joint. Exploration showed an intact FDS tendon of the little finger but a retracted FDP tendon.

Case V

A 34-year-old female received a home-related injury to her hand while opening a door by pushing its sharp edge. The damage resulted in a skin laceration to the palm of her dominant right hand's little finger. During the examination, the patient could flex her fingers at the proximal and distal IP joints except for the little finger at the distal IP joint. Exploration showed an intact FDS tendon of the little finger but a retracted FDP tendon.

Summary of cases

**Figure 1:**
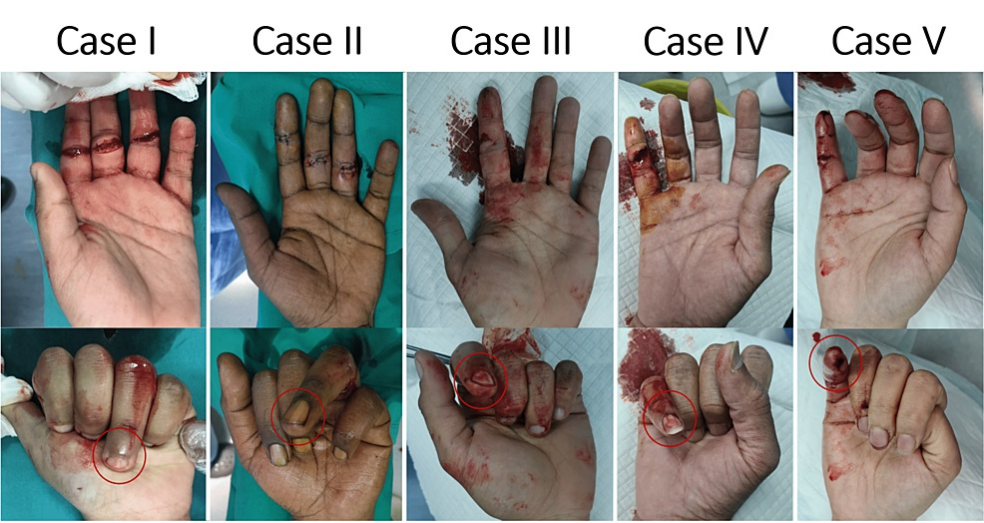
Clinical presentation of the patients Since the tendon attaches to the base of the distal phalanx, the action of the FDP muscle is to flex the finger at the DIP joint. The red circle indicated the injured finger where the lacerated FDP tendon presents. The indicated finger could not be flexed at the DIP joint due to an FDP tendon cut. FDP: flexor digitorum profundus; DIP: distal interphalangeal

Table 1 demonstrates the patients' demographic data while the clinical findings are illustrated in Figure 1. Plain radiographs were devoid of any foreign bodies or bone fractures. No associated injuries to the underlying neurovascular structures were found. The diagnosis of isolated FDP tendon was made following the examination approach listed in the next section. The wounds were cleaned with sodium chloride 0.9% solution, and the skin was then closed with 3-0 non-absorbable polypropylene sutures. The patients were referred to a hand orthopedic surgery unit for further management and care.

**Table 1:** Demographic data of the patients FDP: flexor digitorum profundus

	Case I	Case II	Case III	Case IV	Case V
Age	7	33	24	44	34
Gender	Male	Male	Male	Male	Female
Mechanism of injury	Food-related; Knife’s edge	Food-related; Knife’s edge	Food-related; Knife’s tip	Work-related; Metal’s edge	Home-related; Metal’s edge
Object direction	Sliding inwards	Sliding inwards	Stabbing outwards	Hitting outwards	Hitting outwards
Dominant hand	Right	Right	Right	Right	Right
Injures hand	Left	Left	Left	Right	Right
Injured fingers	Index, Middle, Ring	Index, Middle, Ring	Index	Little	Little
Severed tendon	FDP of the left ring finger	FDP of the left ring finger	FDP of the left index finger	FDP of the right little finger	FDP of the right little finger

## Discussion

Anatomy

Flexor Tendons of the Hand

Flexion of the medial four fingers is achieved by the contraction of the flexor digitorum superficialis (FDS) and profundus (FDP) muscles. Fingers can be flexed individually or in combination with the others, according to the inter-tendinous connections. The FDS flexes the middle phalanges at their proximal interphalangeal (IP) joints while the FDP flexes the distal phalanges at their distal IP joint.

Flexor Zones of the Hand

**Figure 2:**
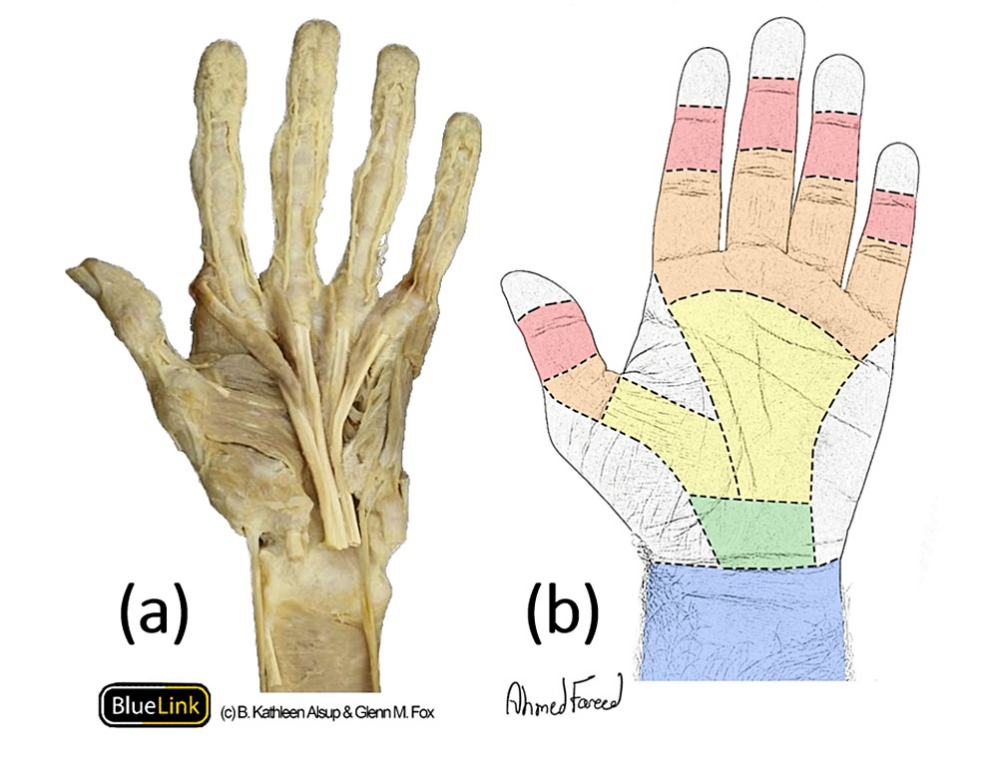
Flexor tendons of the hand and their representative five zones (a) Cadaveric dissection showing the flexor tendons of the left hand (Image courtesy of © B. Kathleen Alsup & Glenn M. Fox; used with permission). (b) Surface anatomy of the flexor zones (zone I: red, zone II: orange, zone II: yellow, zone IV: green, and zone IV: blue; Drawing courtesy of the first author, Ahmed F Alkandari).

There are five zones for the flexor tendons of the hand, as illustrated in Figure 2. Starting distally, zone I begins from the insertion of the FDP to the base of the distal phalanx to the insertion of FDS into the sides of the middle phalanx, where zone II begins. Zone II, the no man's land, ends at the proximal end of the pulley system where zone III begins. Zone III, the lumbrical region, ends at the distal end of the carpal tunnel, where zone IV begins. Zone IV, the carpal tunnel region, ends at the distal end of the carpal tunnel, where zone V begins. From there, zone V extends to the musculotendinous junction in the proximal forearm. Further subdivisions of the first two zones are described in the literature.

Flexor Zone II of the Hand

**Figure 3:**
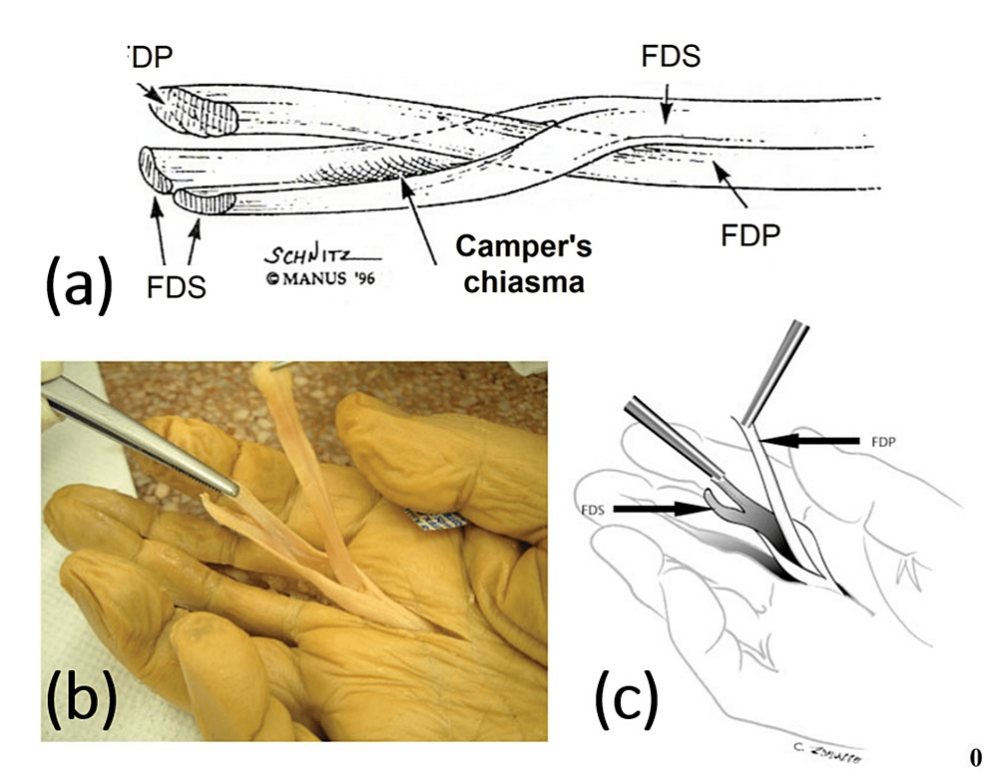
Relation between the FDP and FDS tendons (a) Diagram showing the FDP tendon passing through the Camper's chiasma to be superficial to the FDS tendon in flexor zone II (Copyrighted by Gary Schnitz and the Indiana Hand to Shoulder Center; used with permission). (b,c) Cadaveric dissection of the right middle finger with an illustrative image (Courtesy of © Thieme Medical Publishers; used with permission). The FDP tendon passes through the Camper's chiasma to be inserted on the base of the distal phalanx. The FDS tendon divides and passes around the FDP tendon to be inserted on the lateral sides of the middle phalanx. FDS: flexor digitorum superficialis; FDP: flexor digitorum profundus

In zone II, the FDS tendon splits into the radial and ulnar slips before inserting into the sides of the middle phalanges of the medial four fingers. The FDP tendon passes between the two slips of FDS through Camper's chiasma, inserted into the bases of the distal phalanges of the medial four fingers. The relation between FDS and FDP is shown in Figure 3.

Pulley System of the Flexor Tendons of the Hand

**Figure 4:**
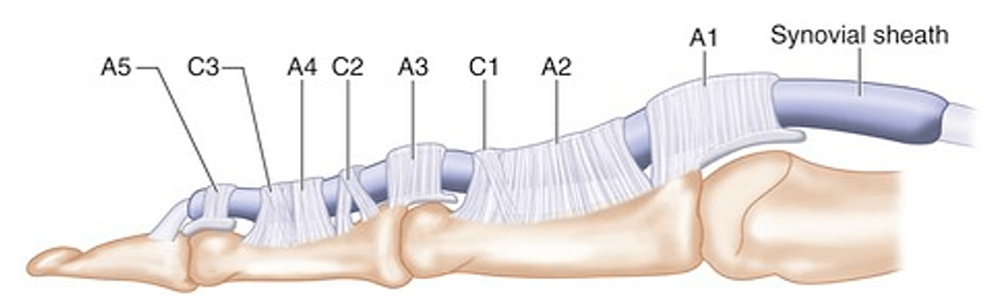
Pulley system of flexor tendons of the hand The pulley system consists of five annular (A1 through A5) and three cruciate (C1 through C3) pulleys that fix the tendons to the phalangeal bones (Courtesy of © Elsevier; used with permission). CDA: common digital artery; VLS: vinculum longus superficialis; TDA: transverse digital artery

The hand's flexor tendons' synovial sheath is reinforced by a fibrous pulley system that keeps them close to the bones, allowing complete flexion of the fingers. The pulley system of the medial four fingers is shown in Figure 4. It consists of five annular (A1 through A5) and three cruciate (C1 through C3) pulleys. In contrast to the flexor zones, the pulley system's enumeration begins proximally.

Vincular System and Blood Supply of FlexorTendons of the Hand

**Figure 5:**
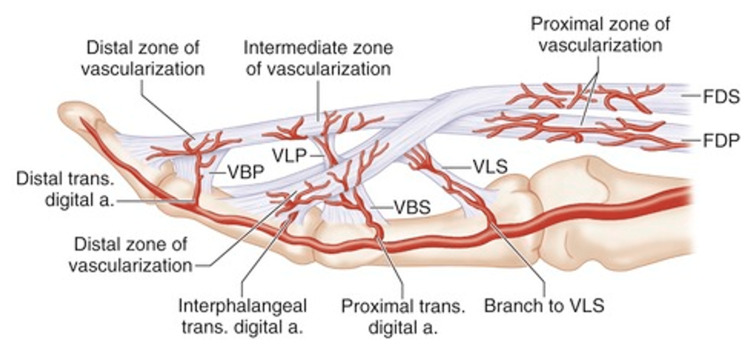
Vincular system and blood supply of the hand The palmar aspect of the flexor tendon has an inadequate blood supply compared to the dorsal one, which affects tendon healing (Courtesy of © Elsevier; used with permission). FDP: flexor digitorum profundus; VLP: vinculum longus profundus; VBP: vinculum brevis profundus; FDS: flexor digitorum superficialis; VLS: vinculum longus superficialis; VBS: vinculum brevis superficialis

The synovial sheet provides the flexor tendon with nutrition and vascularization. While nutrition is provided via passive diffusion through synovial fluid, blood reaches the flexor tendons from digital arteries through connections known as vincula. Each vinculum enters the tendon on its dorsal surface, resulting in the tendon's dorsal aspect being the most well-vascularized. In contrast, the palmer aspect is poorly vascularized and derives its nutrition mainly from the synovial fluid [15]. The blood supply of the flexor tendons of the hand is illustrated in Figure 5.

Case discussion

Mechanism of Injury of Isolated FDP Cut

The demographic data of the five patients are presented in Table 1. It shows that the first three cases were right-dominant males, with a food-related injury caused by a knife that affected the palm of their left non-dominant hands. The difference between the injured fingers in the first two cases and the third one was related to the knife direction, whether sliding inwards or stabbing outwards, respectively. The last two cases were left dominant, with work and home-related injuries that affected their right dominant hands. The little finger was favored because of being the lateral outermost finger while the hand is in an operating supine position. Although the five cases' injuries were in flexor zone II, where both FDS and FDP tendons were present, the severing was isolated to the FDP tendon. The severed FDP tendon of the first two cases was not coincidently confined to the ring finger of their hands. This is due to the flexor cascade phenomenon, which is discussed below.

In the first two cases, the patients used the palm of their left (non-dominant) hands to hold the fruits, cutting with a knife’s edge. Although the three middle fingers (index, middle, and ring) were lacerated, only the ring finger received a deep laceration. Naturally, the little finger is the first to be flexed while making a fist followed by the rest of the fingers, and the last to be extended while supinating the palm preceded by the rest of the fingers. The spinal reflex will react after receiving a knife-cutting injury, causing a rapid flexion of the fingers. This physiological sequence makes the medial fingers more vulnerable to being injured in a knife-cutting mechanism than the lateral ones. The deep wound resulted in the FDP tendon cut in the ring finger of both cases. The FDS tendon was not injured because it splits into radial and ulnar slips before its insertion into the middle phalanx's sides. In the third case, the patient also used his left (non-dominant) hand to hold the frozen burger patty, which he was separating with a knife’s tip. The skin laceration was confined to the palmar aspect of the index finger, which resulted in an isolated FDP tendon cut since the FDS tendon splits into two slips in flexor zone II. In the last two, the patient injured themselves while operating with sharp metal edges. The lacerations were located in the palmar aspect of the little finger of their dominant right hands, which resulted in an isolated FDP tendon cut since the FDS tendon splits into two slips in flexor zone II.

Our findings were consistent with those of Earley and coauthors where hand dominance played a role in determining the wound’s location [4]. Chang and Tay (2018) found that work-related injuries involving the flexor zone II mostly cause lacerations of a single finger of the non-dominant hand [3]. Sikora et al. (2013) reported that pediatric flexor tendon injuries usually involve the dominant hand by manipulating an object (glass or knife) [5]. Further researchers should take a detailed history rather than broadly describing the mechanism of injury. A detailed history should entail the patient’s occupation, the patient’s dominant hand, the causative object and whether it was held by the dominant or non-dominant hand, the direction of the causative object, and the position of the hand during the injury. In our cases, food-related injuries (Case I, II, and III) resulted in wounds located in the non-dominant hands, clearly because the dominant hands were holding the knife. Whereas work-related injury (Case IV) resulted in a wound located in the dominant hand.

Misdiagnosis of Isolated FDP Cut

Tendon injuries are often misdiagnosed and under-managed, compared to other acute injuries. Failure to perform a comprehensive clinical examination is the most likely reason that missed hand injuries occur [16]. Other factors that can contribute to an isolated FDP laceration misdiagnosis, include; the perioperative patient's anxiety; the intact FDS tendon; and the retracted FDP tendon. Successful management in the emergency department (ED) requires effective relief of pain and stress. Insufficiently relaxed hands due to pain or anxiety can make clinical examination difficult, allowing a chance of misdiagnosis. The isolated FDP tendon cut will be retracted and found in the palm away from the wound, causing misdiagnosis. In this case, the intact FDS tendon can cause partial flexion of the distal phalanx since it contributes to some strength of the grip, leading to misdiagnosis too. The functional outcome for tendon injuries in zone II is the worst, partially due to the increased incidence of adhesion formation [7]. Misdiagnosis, in turn, will delay the management and increase the risk of major complications [17]. If left untreated, flexor tendon repair in zone II can lead to a secondary complication resulting in fixed deformities and decreased function [6]. Animal models suggest that the earlier flexor tendons are repaired, the better the patient's functional outcome [10]. While the definitive diagnosis is made by surgical exploration, this will almost always require wound extension. A skillful hand surgeon should design this wound extension to anticipate adhesions and other complications [12].

Examination approach to detect flexor tendon injuries

History Taking

The mechanism of injury is an essential element for the diagnosis of tendon laceration, as well as for defining the optimal treatment [7]. Patient occupation and hand dominance can give a clue about the instrument and the wound.

Physical Examination

**Figure 6:**
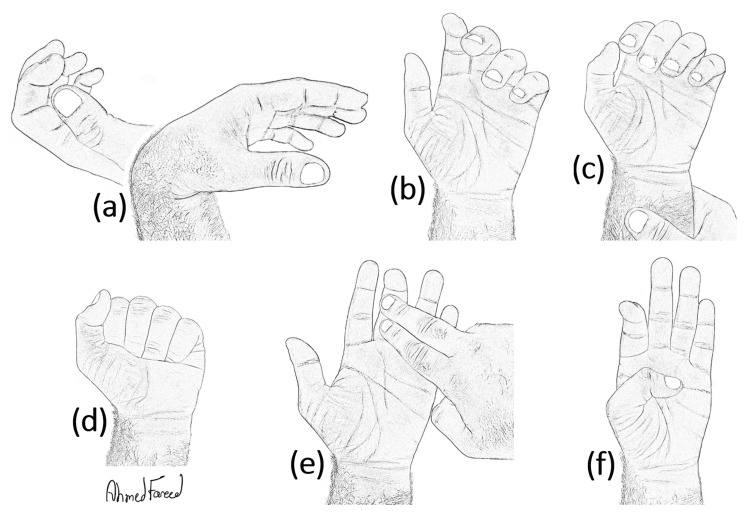
Physical examination to diagnose an isolated FDP tendon cut (a) Tenodesis phenomenon. (b) Cascade phenomenon. (c) Forearm compression test. (d) Combined FDS and FDP test. (e) Independent FDP test. (f) Linburg-Comstock test. (Drawing courtesy of the first author, Ahmed F Alkandari)

Comparing the injured hand to the contralateral normal one is essential to detect abnormal positioning. While the fingers are resting, the wrist's flexion will normally cause the fingers to extend, and the wrist's extension will cause them to flex. This phenomenon is called the tenodesis effect [18], which will be absent in case of a tendon cut. While the wrist is in a neutral position, the fingers will be aligned with different flexion degrees at all their joints, beginning with less flexion at the index and progressing to more flexion towards the little finger. This is called the cascade phenomenon [19], which may be disrupted if a flexor tendon laceration occurs. However, since the FDS tendon contributes to some strength of the grip, an injured FDP tendon may not completely disrupt the cascade. Like the Simmonds-Thomspon test of Achilles tendon rupture, the action of flexor muscles of the hand can be tested by exerting pressure on the forearm. The forearm compression test will cause all fingers to flex when the flexor tendons are intact. The absence of flexion in a finger denotes an injured flexor tendon. As shown in Figure 1, if the FDP tendon is completely lacerated, the unsupported finger will assume an extended position at the distal IP. This test allows the physician to examine the action of both FDS and FDP tendons, however, this test can be painful without local anesthesia. A physician's attempt to extend the distal phalanx of each flexed finger can show the integrity of the FDP tendon. A lacerated FDP tendon will cause a loose distal phalanx while doing so. To examine the function of the FDP tendon independently, immobilizing the middle phalanx while forcibly hyperextending the distal phalanx will allow an individual evaluation of FDP action. The examiner should immobilize the injured finger's middle phalanx to neutralize any input from the FDS tendon. A patient who cannot flex his/her distal phalanx at the distal IP joint must be suspected to have a severed FDP tendon. However, this maneuver should be performed under local anesthesia to ensure a full range of motion. Linburg-Comstock variation, found in 31% of the population, comprises a tendon slip connecting the FPL and the FDP tendon, usually of the index finger [20]. When it presents, flexion of the thumb's distal phalanx will result in a simultaneous flexion of the distal phalanx of the index. This can be used to test the FDP tendon's integrity in the traumatic lesion on zone II of the index finger. A summary of the physical examination is illustrated in Figure 6.

Surgical Exploration

Using a tourniquet is needed to dry the surgical field and improve the identification of normal and injured structures. Wound irrigation is required to ensure a clear surgical field, especially with contaminated and dirty wounds. Wound dissection should begin in the non-injured area for better orientation and proceed toward the injury site [12]. Wound extension should be done by a skilled hand surgeon.

## Conclusions

Using a systematic examination approach for traumatic hand injuries is essential to ensure a proper assessment. Injuries to flexor zone II should be kept in mind to avoid misdiagnosing an isolated FDP tendon laceration. Understanding the mechanism of injury, together with basic anatomical knowledge of flexor tendons of the hand, should allow ED physicians to identify tendon injuries, anticipate complications, and provide effective healthcare.
